# Association between Pseudoexfoliation and Alzheimer's disease‐related brain atrophy

**DOI:** 10.1002/alz70855_099546

**Published:** 2025-12-23

**Authors:** Jeong Ho Han, Heewon Bae, Eun Joo Choi

**Affiliations:** ^1^ Department of Neurology, Veterans Health Service Medical Center, Seoul, Korea, Republic of (South); ^2^ Veterans Health Service Medical Center, Seoul, Korea, Republic of (South); ^3^ Evergreen Neuropsychiatry clinic, Seoul, Korea, Republic of (South)

## Abstract

**Background:**

Pseudoexfoliation(PEX) syndrome is an age‐related disorder of the extracellular material accumulation in the anterior segment of eye. PEX material is not clearly known, but several studies have found amyloid as a PEX component. Alzheimer's disease(AD) is a representative disorder in which amyloid accumulates. The process of deposition of PEX material has similar features to the amyloid aggregation of AD. Amyloid deposition causes brain atrophy in AD. This study investigated whether PEX syndrome associated with brain atrophy and AD.

**Method:**

We reviewed the medical records of patients diagnosed with PEX between January 2015 and August 2021 at the Veterans Health Service Medical Center. This retrospective cohort study involved 48 patients with PEX and 48 healthy age‐ and gender‐ matched control participants. PEX patients were divided into 2 groups with or without glaucoma. The main outcome measure was the brain atrophy using visual rating scale and incidence of AD.

**Result:**

The participants with medial temporal atrophy were 56.3% for PEX, 35.4% for control group. The global cortical atrophy and parietal atrophy was significantly higher in the PEX group. (*p* <0.05) Comparison between the PEX syndrome and PEX glaucoma showed no difference in brain atrophy. Among the 96 participants, those diagnosed with AD were 16 and 5 participants in PEX and control group, respectively. PEX glaucoma tended to have lower MMSE score than those without glaucoma.

**Conclusion:**

PEX is associated with brain atrophy, which reflect the AD risk. PEX glaucoma may show advanced stages of AD. Our studies suggest that PEX can be a predictor of early phase of AD.

